# Neurogenic Potential of the 18-kDa Mitochondrial Translocator Protein (TSPO) in Pluripotent P19 Stem Cells

**DOI:** 10.3390/cells10102784

**Published:** 2021-10-17

**Authors:** Laura González-Blanco, Juan Carlos Bermejo-Millo, Gabriela Oliveira, Yaiza Potes, Eduardo Antuña, Iván Menéndez-Valle, Ignacio Vega-Naredo, Ana Coto-Montes, Beatriz Caballero

**Affiliations:** 1 Department of Morphology and Cell Biology, Faculty of Medicine, University of Oviedo, Julián Clavería s/n, 33006 Oviedo, Spain; laurablanco94@hotmail.com (L.G.-B.); bermejomillo@gmail.com (J.C.B.-M.); potesyaiza@uniovi.es (Y.P.); edugan.97@gmail.com (E.A.); ivan_vallinas@hotmail.com (I.M.-V.); vegaignacio@uniovi.es (I.V.-N.); acoto@uniovi.es (A.C.-M.); 2 Instituto de Investigación Sanitaria del Principado de Asturias (ISPA), 33011 Oviedo, Spain; 3 Instituto de Neurociencias del Principado de Asturias (INEUROPA), 33006 Oviedo, Spain; 4 CNC-Center for Neuroscience and Cell Biology, University of Coimbra, 3060-197 Cantanhede, Portugal; glopesoliv@gmail.com

**Keywords:** TSPO, mitochondria, stem cells, neurogenesis

## Abstract

The 18-kDa translocator protein (TSPO) is a key mitochondrial target by which different TSPO ligands exert neuroprotective effects. We assayed the neurogenic potential of TSPO to induce the neuronal differentiation of pluripotent P19 stem cells in vitro. We studied changes in cell morphology, cell proliferation, cell death, the cell cycle, mitochondrial functionality, and the levels of pluripotency and neurogenesis of P19 stem cells treated with the TSPO ligand, PK 11195, in comparison to differentiation induced by retinoid acid (RA) and undifferentiated P19 stem cells. We observed that PK 11195 was able to activate the differentiation of P19 stem cells by promoting the development of embryoid bodies. PK 11195 also induced changes in the cell cycle, decreased cell proliferation, and activated cell death. Mitochondrial metabolism was also enhanced by PK 11195, thus increasing the levels of reactive oxygen species, Ca^2+^, and ATP as well as the mitochondrial membrane potential. Markers of pluripotency and neurogenesis were also altered during the cell differentiation process, as PK 11195 induced the differentiation of P19 stem cells with a high predisposition toward a neuronal linage, compared to cell differentiation induced by RA. Thus, we suggest a relevant neurogenic potential of TSPO along with broad therapeutic implications.

## 1. Introduction

The mitochondrial 18-kDa translocator protein (TSPO), which was first described as the peripheral benzodiazepine receptor, is primarily located in the mitochondrial outer membrane in different cell types and tissues. However, TSPO is particularly abundant in steroidogenic organs, such as the testis and ovary [1,2,3], where it participates in cholesterol translocation into the mitochondria for the synthesis of pregnenolone (a precursor of steroid hormones). In addition, TSPO is involved in the generation of reactive oxygen species (ROS), regulation of Ca^2+^ levels, ATP production, immune and inflammatory responses, and processes of cell death and cell proliferation [2,3,4,5,6]. Recently, a relevant function of TSPO in the regulation of nuclear gene expression was described [7], which may explain the variety of functions in which TSPO is involved.

The central nervous system (CNS) has low levels of TSPO that are restricted to glial cells [8]. Notably, TSPO expression is significantly increased in glial cells under neurodegeneration and inflammation conditions, including neurodegenerative pathologies such as Parkinson’s and Alzheimer’s diseases as well as after traumatic brain injury (TBI) [6]. Thus, TSPO is commonly used in clinical practice as a biomarker for monitoring neuroinflammation with positron emission tomography (PET) in patients with several neurological and psychiatric conditions [6]. Increased TSPO levels have also been observed in blood from patients with TBI, which correlate with systemic and soluble proinflammatory parameters (e.g., IL-6, TNF-α, reactive C protein) as well as the level of neurological activity [9], thus it represents a valuable marker for monitoring the prognosis of brain injury. Interestingly, damaged neurons also show increased TSPO levels in those specific zones affected by brain injury [10], as well as under cell stress conditions in zones such as the brain cortex and hippocampus [11].

The neuroprotective benefit of treatments with TSPO ligands has been documented in multiple in vitro and in vivo studies, thus suggesting that TSPO represents a potent therapeutic target against neurodegenerative and neuroinflammatory lesions [5,12,13,14]. In particular, treatments with different TSPO ligands, such as PK 11195, inhibited cell death, the inflammatory response and processes of excitotoxicity in different in vitro and in vivo study models and under different toxic and neurodegenerative conditions (e.g., glutamate, kainic acid, and ammonium chloride) [5,7,12,13,15]. For instance, PK 11195 decreases the proinflammatory response of the reactive microglia activated by lipopolysaccharide in vitro [6,15] as well as being capable of reducing microglial activation in the rats striatum after the administration of quinolinic acid [16]. PK 11195 is also able to regulate cell proliferation, ROS generation, Ca^2+^ levels, ATP synthesis, mitochondrial membrane potential (∆Ψm), and gene expression [5,7,12,13,15]. Other TSPO ligands also have relevant neuroprotective effects, reducing inflammation and oxidative damage, attenuating mitochondrial dysfunction, and preventing cell death after a neurodegenerative injury [13,17,18]. Additionally, recent studies have observed that TSPO ligands induce neuronal differentiation of various cell types in vitro. In this way, PK 11195 induces neuronal differentiation of PC12 and N1E-115 cells [7,19]. Common structural features are necessary for the efficacy of TSPO ligands, such as PK 11195 (an isoquinolinecarboxamide). The receptor binding site provides a hydrogen bond-donating function (H_1_) and three lipophilic pockets (L_l_, L_2_, and L_3_): L_1_ is considered the central core and it always includes an aromatic ring; L_2_ includes an aryl ring and it may sometimes have a halogen or a second aromatic ring; and L3 is an amide link always presented outside the carbocycle in PK 11195 [5].

Several preclinical studies based on mesenchymal or neuronal stem cell transplantation have reported a promising approach for therapies against brain injury [20]. Transplanted stem cells are capable of self-renewal and differentiation into both neurons and glial cells, promoting the release of cytokines and neurotrophic growth factors, which allow the recovering of structural and functional plasticity of the damaged nervous tissue [21]. Therefore, in addition to the well-known neuroprotective effects of TSPO ligands, which regulate different essential cell functions—including cell death and cell proliferation processes, among others (e.g., ATP production, Ca^2+^ levels, ROS generation, ∆Ψm, etc.) [2,3,4,5,6,7]—the application of TSPO ligands at more appropriate concentrations might activate a regulatory mechanism able to induce the differentiation of stem cells. The present study supports the valuable neurogenic potential of TSPO and the use of their binding ligands to induce the neuronal differentiation of embryonal P19 pluripotent stem cells, with broad therapeutic implications. These findings acquire special relevance in tissues with little or no cell repair capacity, such as damage to the CNS.

## 2. Materials and Methods

### 2.1. Cell Culture and Treatments

Murine P19 embryonal carcinoma cells were obtained from the American Type Culture Collection (ATCC^®^, CRL-1825). They are pluripotent embryonic stem cells that are able to differentiate in vitro into multiple cell types, including cardiomyocytes, muscle skeletal cells, glial cells, and neurons [22]. Undifferentiated P19 stem cells were cultured in a monolayer at 37 °C with 5% CO_2_ in high-glucose Dulbecco’s modified Eagle’s medium (DMEM) supplemented with 10% fetal bovine serum (FBS), 110 mg/L sodium pyruvate, 1.8 g/L sodium bicarbonate, and a 1% solution of an antibiotic-antifungal agent. Undifferentiated P19 stem cells were maintained in a monolayer with passages every 2–3 days at a 1:20 dilution. Undifferentiated P19 stem cells were seeded in plates at a density of 3 × 10^5^ cells/cm^2^ and cultured under three experimental conditions for 4 days to induce cell differentiation: (a) undifferentiated P19 stem cells (SC); (b) P19 stem cells treated with a single dose of 1 µM Retinoic Acid (RA); and c) P19 stem cells treated with a single dose of the TSPO ligand PK 11195 at 50 µM (PK 11195), as described in previous studies [7]. Cell morphology was evaluated during the differentiation of P19 stem cells using a phase contrast light microscope (Nikon Eclipse TS100) and the NIS Elements F v4.60 software.

### 2.2. Western Blot Analysis

Adherent P19 stem cells were harvested, resuspended in RIPA buffer supplemented with 2 mM dithiothreitol (DTT) and a protease inhibitor cocktail, and physically ruptured by sonication. The protein concentration was quantified according to the Bradford method [23]. Twenty micrograms of protein were electrophoretically separated on SDS-polyacrylamide gels and transferred to polyvinylidene difluoride (PVDF) membranes at 350 mA. Membranes were stained with 0.1% Ponceau S to ensure equal loading (load control). After blocking the membranes with 10% nonfat dry milk in TBS-T (50 mM Tris-HCl, pH 7.5; 150 mM NaCl and 0.1% Tween-20) for 1 h at room temperature, membranes were incubated overnight at 4 °C with the following primary antibodies: TSPO (PA5-75544, Invitrogen), Oct4-A (Octamer-binding transcription factor 4A, 2840, Cell Signaling), Sox2 (Sex determining region Y-box2, 2748, Cell Signaling), Nanog (Homeobox protein Nanog, ab80892, Abcam), Troma-1 (Cytokeratin 8, Developmental Studies Hybridoma Bank), Nestin (Neuroectodermal stem cell protein, N5413, Sigma), BLBP (Brain lipid binding protein, ABN14, Sigma), Msi1 (Musashi RNA-binding protein 1, M3571, Sigma), Mash1 (Mammalian achaete-scute homolog 1, 14-5794-82, Invitrogen), Tbr2 (T-box brain gene 2, ABN1687, Sigma), NeuroD1 (Neurogenic differentiation 1, PA5-11889, Invitrogen), Dcx (Doublecortin, D9693, Sigma), NeuN (Neuronal nuclear antigen, AB4301175, Sigma), TUBB3 (β-III-Tubulin, sc-80005, Santa Cruz Biotechnology), each one previously diluted 1:1000 in blocking buffer (1% nonfat dry milk in TBS-T). After three 5-min washes with TBS-T, the membranes were incubated with the corresponding horseradish peroxidase conjugated secondary antibody diluted 1:5000 in TBS-T containing 1% (w/v) nonfat dry milk for 1 h at room temperature, followed by three 10-min washes with TBS-T. The membranes were developed using a chemiluminescent substrate (WBKLS0500, Merck Millipore), according to the manufacturer’s protocol. The optical density was quantitated using the Image Studio Lite 5.2.5 software (LI-COR Biotechnology). The results from at least three independent cultures and treatments were normalized to the loading control (ponceau S) and expressed as relative percentages to undifferentiated P19 stem cells (SC). We should note that the complete ponceau S staining for each antibody is shown in the Supplementary Figure S1. The entire membrane of ponceau S was used for total protein normalization for each specific antibody.

### 2.3. Cell Cycle

The cell cycle profile was analyzed based on the cellular DNA content. One million adherent P19 stem cells were harvested, washed with PBS and fixed by adding 70% cold (−20 °C) ethanol. Subsequently, the cells were centrifuged at 850 g to remove the ethanol, washed twice with PBS and finally resuspended in 400 μL of PBS. Propidium iodide (20 μg/mL, P4864, Sigma-Aldrich) and 10 µg/mL RNase cocktail (R5000, Sigma-Aldrich) were added and incubated for 30 min at 37 °C. A total of 20 × 10^3^ events per sample were analyzed using a Cytoflex S flow cytometer (Beckman Coulter, CA, USA) with excitation and emission wavelengths of 488 nm and 605 nm, respectively. All experimental conducted with a voltage of 673 and the FL3 detector with a bandpass of 602–628. The percentages of cells in G_1_/G_0_, S, and G_2_/M phases were determined using the CytExpert 2.1 software.

### 2.4. Cell Proliferation

Cell proliferation was measured using the sulforhodamine B (SRB) assay. P19 cells were seeded on 96-multiwell plates at a cellular density of 5 × 10^3^ cells/mL. At 0, 24, 48, 72, and 96 h after seeding, the medium was removed and the wells were rinsed with 1% PBS. Adherent cells were then fixed overnight with ice-cold 1% (v/v) acetic acid in methanol at −20 °C. The fixation solution was then discarded, and the plates were dried in an oven at 37 °C. Two hundred and fifty microliters of 0.5% SRB in a 1% acetic acid solution were added and incubated at 37 °C for 1 h. Afterwards, the wells were washed with 1% acetic acid in water and dried. Two hundred milliliters of Tris buffer (pH 10) were added, and the plates were shaken for 30 min before measuring the optical density at 540 nm, with a reference filter of 690 nm.

### 2.5. Live/Dead Cell Assay

An Annexin V-fluorescein isothiocyanate (FICT) apoptosis detection kit (APOAF, Sigma-Aldrich) was used to determine the percentages of viable and dead cells. Briefly, both adherent and DMEN-floating P19 cells were washed twice with cold PBS and resuspended in 250 µL of binding buffer at a cell density of 2.5 × 10^5^ cells/mL. Then, 5 µL of Annexin V-FICT and 10 µL of PI solution were added to the cells and incubated in dark at room temperature for 15 min. The fluorescence of at least 20 × 10^3^ cells was analyzed using a Cytoflex S flow cytometer (Beckman Coulter, CA, USA) with an excitation wavelength of 488 nm. The simultaneous measurement of Annexin-V-FICT/PI fluorescence was performed using a 530 nm bandpass filter for annexin-V-FICT and a 610/20 nm bandpass filter for PI fluorescence. Cells that are early in the apoptotic process will be stained with the Annexin-V-FICT conjugate alone. Cells in late apoptosis will be stained with both PI and the Annexin-V-FICT conjugate. Necrotic cells will be stained with PI alone. Live cells will show no staining for either PI or the Annexin-V-FICT conjugate. The percentages of cells in necrosis, early apoptosis, and late apoptosis were determined using the CytExpert 2.1 software.

### 2.6. Mitochondrial Membrane Potential (∆Ψm)

Changes in the ∆Ψm were evaluated using the MitoProbe TMRM (tetramethylrhodamine methyl ester) Assay Kit (M20036, Thermo Fisher Scientific). TMRM is a lipophilic cationic dye that accumulates in polarized mitochondria, while its fluorescence is diminished after mitochondrial membrane depolarization. Briefly, adherent P19 stem cells at a density of 1 × 10^6^ cells/mL were incubated with 20 nM TMRM for 30 min at 37 °C and 5% CO_2_. Then, the dye was removed and the cells were resuspended in 500 μL of PBS. Fluorescence was measured using a Cytoflex S flow cytometer (Beckman Coulter, CA, USA) at excitation and emission wavelengths of 561 and 585 nm, respectively.

### 2.7. Mitochondrial Calcium Level Measurement

The mitochondrial Ca^2+^ content was assayed in lived P19 stem cells with the fluorescent Ca^2+^ indicator Rhod-2-AM (R1245MP, Invitrogen). Briefly, 3 × 10^4^ cells were seeded in an open μ-Slide (chambered coverslip) of eight wells (80826, Ibidi, Martinsried, Germany) and incubated in the dark with 5 μM Rhod-2-AM for 60 minutes at 37 °C. After two washes with Hank’s Balanced Salt Solution (HBSS, 14025092, Gibco), the fluorescence signal from these cells was measured using the Leica TCS-SP8X confocal microscope, with excitation and emission wavelengths of 552 and 581 nm, respectively.

### 2.8. Reactive Oxygen Species (ROS) Measurement

The generation of ROS in P19 stem cells was investigated by measuring the fluorescence of 2′,7′-dichlorofluorescin diacetate with a thiol-reactive chloromethyl group (CM-H_2_DCFDA, C6827, Invitrogen, CA, USA), which covalently binds to intracellular components, permitting even longer retention within the cell. A fresh stock solution of CM-H_2_DCFDA was prepared in DMSO and diluted to a final concentration of 10 µM in HBSS (14025092, Gibco). A total of adherent 1 × 10^6^ cells/mL were harvested and washed with PBS before adding 500 µL of the working solution with CM-H_2_DCFDA for incubation in the dark at 37 °C for 60 min. Then, the cells were washed with HBSS twice and fluorescence was measured using a Cytoflex S flow cytometer (Beckman Coulter) at excitation and emission wavelengths of 492 and 530 nm, respectively. The negative control was a dye-free mixture of cells.

### 2.9. ATP Measurement

The adenosine 5′-triphosphate (ATP) bioluminescent assay kit (FLAA, Sigma-Aldrich) was used to determine the intracellular levels of ATP, according to manufacturer’s instructions. The assay measured the light emission with a SIRIUS luminometer (Berthold, Pforzheim, Germany) based on the ATP consumption when luciferase catalyzes D-luciferin oxidation. The concentrations of ATP were expressed as nmol ATP/g protein. The protein concentration was quantified according to the Bradford method [23].

### 2.10. Statistical Analyses

Data are presented as the mean values ± SEM. Multiple comparisons were carried out using one- or two-way ANOVA, as appropriate, followed by Sidak’s multiple comparisons post hoc test. Significance was accepted at *p* < 0.05. The normality of the samples was verified using the Kolmogorov–Smirnov (K–S) test. Statistical analyses and histograms were performed using GraphPad Prism 6 software.

## 3. Results

### 3.1. TSPO Levels in Pluripotent P19 Stem Cells

**Figure 1:**
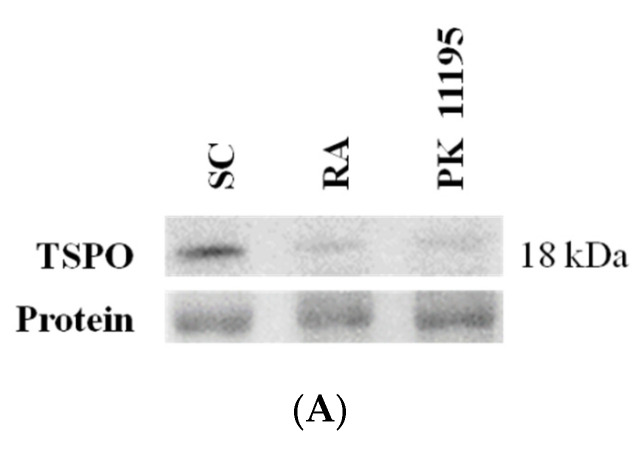

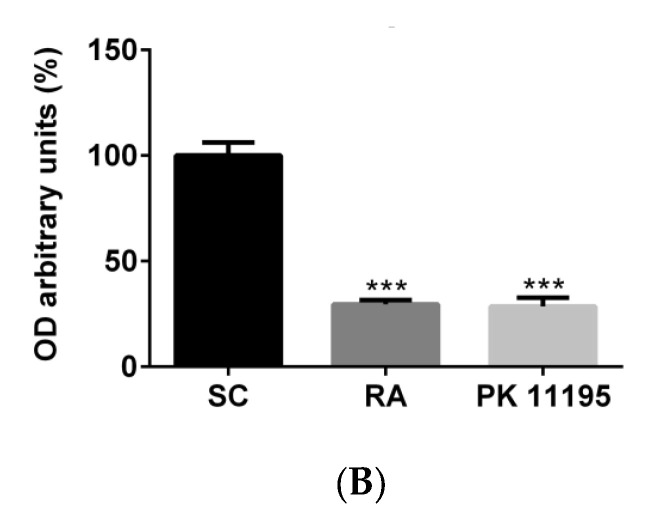
(**A**) Representative immunoblot of the TSPO protein levels in undifferentiated P19 stem cells (SC) and cells treated with 1 μM retinoid acid (RA) or 50 μM PK 11195 (PK 11195) during 4 days of treatment. (**B**). Bar chart shows quantification of the optical densities (OD) from three independent cell cultures and treatments. Data are expressed as means ± SEM. *** *p* < 0.001 *versus* SC. Protein shows a representative ponceau staining of one of the experiments. The complete ponceau staining for TSPO antibody is shown in the Supplementary Figure S1. Although the entire membrane of ponceau S was used for total protein normalization, only a representative section of the membrane is displayed in this image. TSPO, the 18-kDa mitochondrial translocator protein.

PK 11195 is a classical high-affinity TSPO ligand [2,3,4,5,6,7], and given that TSPO expression in P19 stem cells have not been described to date, we first studied TSPO protein levels in undifferentiated P19 stem cells (SC) as well as changes in its expression during the differentiation of P19 stem cells in response to 4-day treatments with RA or PK 11195 (Figure 1A, B). Interestingly, TSPO levels were noticeably expressed in P19 undifferentiated stem cells (SC), while its levels significantly decreased (Figure 1B, *p* < 0.001) in cells treated with RA or PK 11195 compared to undifferentiated P19 stem cells (SC).

### 3.2. Cell Differentiation of P19 Stem Cells by RA or PK 11195

**Figure 2:**
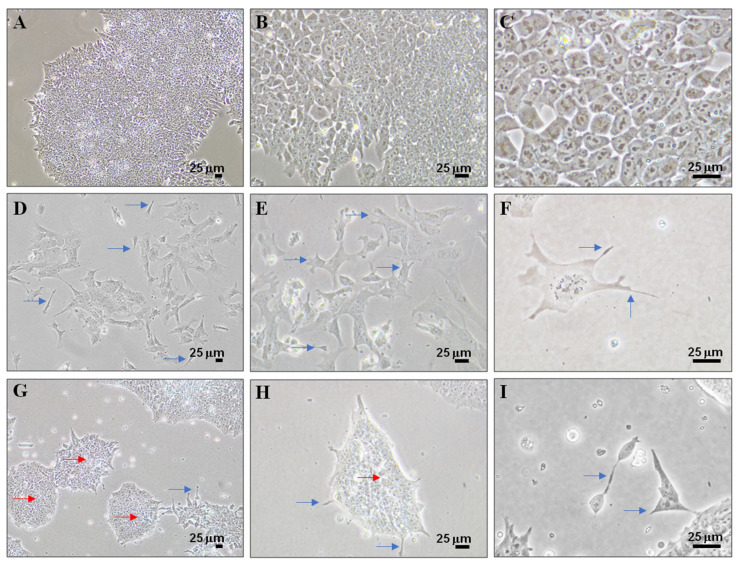
Phase contrast microscopy of undifferentiated P19 stem cells (**A**–**C**) and cells treated with 1 μM retinoid acid (**D**–**F**) or 50 μM PK 11195 (**G**–**I**) during 4 days of culture. Blue arrows show cell extensions; Red arrows show spherical growing colonies. Scale bar at the bottom right, 25 μM. (**A**,**D**,**G**) were taken at 100× magnification; (**B**,**E**,**H**) were taken at 20× magnification; (**C**,**F**,**I**) were taken at 400× magnification.

The morphological differences in P19 stem cells cultures for 4 days under the experimental conditions analyzed in this study (SC, RA, and PK 11195) are shown in Figure 2. Undifferentiated P19 stem cells (SC, Figure 2A–C) grew in monolayer attached to the surface of the plate and at a high cell density after 4 days of culture. Treatment with RA (Figure 2D–F) generated a loss of cell density and the growth of multiple cell extensions (blue arrows) in response to cell differentiation processes. Cell extensions were also observed in PK 11195-treated cells (Figure 2G–I). However, the PK 11195 treatment caused a drastic formation of spherical growing colonies (red arrows) that resembles embryoids bodies (Figure 2G–I).

### 3.3. Pluripotency and Neuronal Markers during the Differentiation of P19 Stem Cells

**Figure 3:**
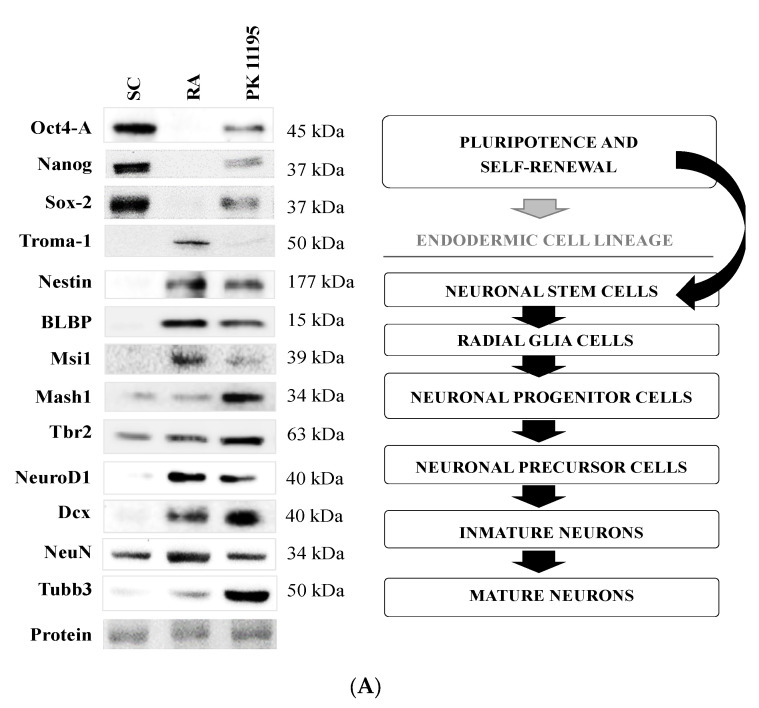

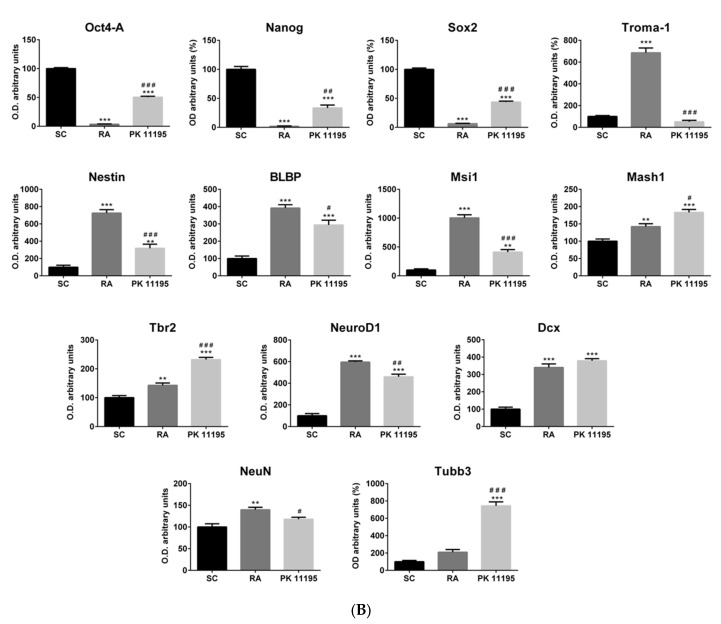
(**A**) Markers of pluripotency and neuronal differentiation in undifferentiated P19 stem cells (SC) and cells treated with 1 μM retinoid acid (RA) or 50 μM PK 11195 (PK 11195) during 4 days of treatment. (**B**) Bar chart shows quantification of the optical densities (OD) from three independent cell cultures and treatments. Data are expressed as means ± SEM. ** *p* < 0.01 *versus* SC; *** *p* < 0.001 *versus* SC; # *p* < 0.05 *versus* RA; ## *p* < 0.01 *versus* RA; ### *p* < 0.001 *versus* RA. Protein shows a representative ponceau staining of one of the experiments. The complete ponceau staining for each antibody is shown in the Supplementary Figure S1. Although the entire membrane of ponceau S was used for total protein normalization, only a representative section of the membrane is displayed in this image.

Recently, it has been demonstrated that PK 11195 may regulate nuclear gene expression [7]. Therefore, we assayed the impact of our treatments (SC, RA, PK 11195) on protein levels of different markers of pluripotency and neuronal differentiation in our P19 stem cells, as is shown in Figure 3A,B). Pluripotency and self-renewal are represented by the levels of Oct4-A, Nanog, and Sox-2 [24], which were significantly decreased in cells treated with RA or PK 11195 compared to undifferentiated P19 stem cells (Figure 3B, *p* < 0.001). However, the levels of Oct4-A, Sox2 (*p* < 0.001), and Nanog (*p* < 0.01) were always higher in PK 11195-treated cells than in RA-treated cells (Figure 3B). The endoderm-specific cytokeratin Troma-1 [25] was mainly expressed in P19 stem cells treated with RA compared to the low levels observed in PK 11195-treated cells (Figure 3B, *p* < 0.001) and in undifferentiated P19 stem cells (Figure 3B, *p* < 0.001). Levels of Nestin, a marker of neural stem cells [24], were always increased in RA-treated (*p* < 0.001) or PK 11195-treated (*p* < 0.01) cells compared to undifferentiated P19 stem cells (Figure 3B). However, lower Nestin levels were detected in PK 11195-treated cells than in RA-treated cells (Figure 3B, *p* < 0.001). The level of BLBP, a radial-glia marker [24], was significantly increased after treating cells with RA or PK 11195 as compared to undifferentiated P19 stem cells (Figure 3B, *p* < 0.001). BLBP expression was also lower in PK 11195-treated cells than in RA-treated cells (Figure 3B, *p* < 0.05). Msi1, Mash1, and Tbr2 were used as markers of neuronal progenitor cells [24,26]. Msi1 expression was significantly increased in cells treated with RA (*p* < 0.001) or PK 11195 (*p* < 0.01) compared to undifferentiated P19 stem cells (Figure 3B). However, much lower Msi1 expression was detected in PK 11195-treated cells than in RA-treated cells (Figure 3B, *p* < 0.001). Mash1 and Tbr2 levels were significantly increased in cells treated with RA (Figure 3B, *p* < 0.01) or PK 11195 (Figure 3B, *p* < 0.001) compared to undifferentiated P19 stem cells. Notably, Mash 1 (*p* < 0.05) and Tbr2 (*p* < 0.001) expression was always higher in PK 11195-treated cells than in RA-treated cells (Figure 3B). The levels of NeuroD1 and Dcx, markers of neuronal precursor cells [24], were significantly increased in cells treated with RA or PK 11195 compared to undifferentiated P19 stem cells (Figure 3B, *p*< 0.001). However, lower NeuroD1 levels were observed in PK 11195-treated cells than in RA-treated cells (Figure 3B, *p* < 0.01). Finally, markers of postmitotic neurons such as NeuN and β-III-Tubulin (Tubb3) [24], were expressed during the differentiation of P19 stem cells. NeuN levels were significantly higher in RA-treated cells compared to undifferentiated P19 stem cells (Figure 3B, *p* < 0.01) and PK 11195-treated cells (Figure 3B, *p* < 0.05). Tubb3 expression was increased in PK 11195-treated cells compared to RA-treated cells and undifferentiated P19 stem cells (Figure 3B, *p* < 0.001).

### 3.4. Cell Cycle during Cell Differentiation of P19 Stem Cells

**Figure 4:**
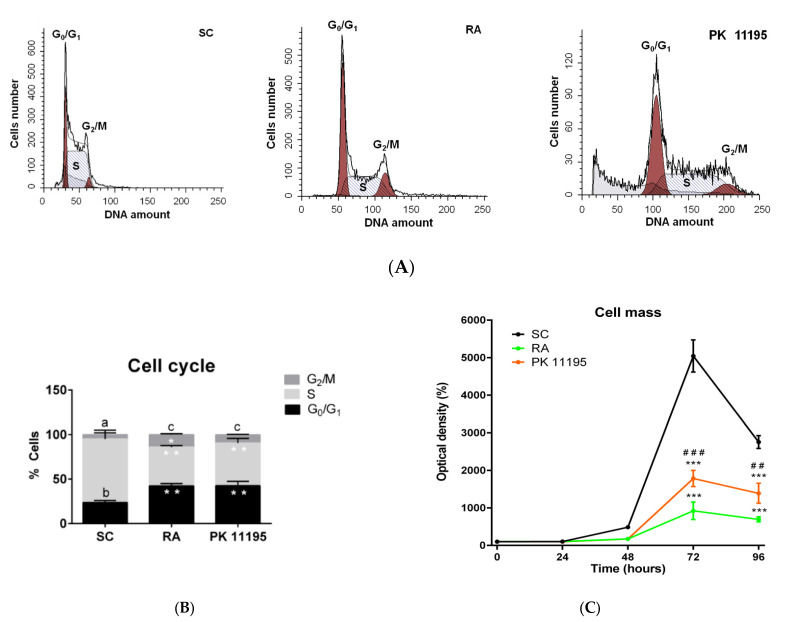
The cell cycle and cell mass in undifferentiated P19 stem cells (SC) and treated with 1 μM retinoid acid (RA) or 50 μM PK 11195 (PK 11195) during 4 days of treatment. (**A**) The cell cycle was analyzed by flow cytometry using propidium iodide. Representative histograms show relative fluorescence of DNA amount respect to cell number for each experimental condition (SC, RA and PK 11195). (**B**) Bar charts represent means ± SEM from the percentage of cells in G_1_/G_0_, S and G_2_/M phase from three independent cultures and treatments. * *p*< 0.05 *versus* SC; ** *p*< 0.01 *versus* SC; ^a^ *p*< 0.001 S *versus* G_0_/G_1_ and G_2_/M in SC; ^b^ *p*< 0.05 G_0_/G_1_ *versus* G_2_/M in SC.; ^c^ *p* < 0.001 G_2_/M *versus* G_0_/G_1_ and S in RA and PK 1195. (**C**) Cell mass was assayed by the SRB assay. Relative SRB absorbance was expressed as percentages respect to SC at 0 h. Data was expressed as means ± SEM from three independent cultures and treatments. *** *p*< 0.001 *versus* SC; ^# #^ *p*< 0.01 *versus* RA; ^# # #^ *p* < 0.001 *versus* RA.

Given that we microscopically observed a loss of cell density in differentiated P19 stem cells (Figure 2), and that PK 11195 can affect cell proliferation [2,3,4,5,6,7], we assayed cell cycle profile and cell mass in our experimental conditions (SC, RA, PK 11195). The percentages of P19 stem cells in different phases of the cell cycle (G_0_/G_1_, S, and G_2_/M) are shown in Figure 4A,B. The percentage of cells in G_0_/G_1_ phase was significantly increased (Figure 4B, *p* < 0.01) in cells treated with RA or PK 11195 compared to undifferentiated P19 stem cells (SC). However, the percentage of cells in S phase was noticeably higher in undifferentiated P19 stem cells than in cells treated with RA or PK 1195 (Figure 4B, *p* < 0.01). The percentage of cells in G_2_/M phase was higher in the treated and differentiated cells than in the undifferentiated P19 stem cells, but the difference was only statistically significant in the RA-treated cells (Figure 4B, *p* < 0.05). Likewise, undifferentiated P19 stem cells showed an increased percentage of cells in S phase (Figure 4B, *p*< 0.001) compared to the percentages of cells in G_0_/G_1_ or G_2_/M phases. The percentage of cells in G_0_/G_1_ phase was also higher than that of cells in G_2_/M phase (Figure 4B, *p*< 0.05). However, P19 stem cells treated with RA or PK 11195 showed the lowest percentage of cells in G_2_/M phase compared to cells in S or G_0_/G_1_ phase (Figure 4B, *p*< 0.001). Curiously, and given the well-known pro-apoptotic effects of PK 11195 at micromolar concentrations [5,12], we observed a relevant population of sub-G_1_ cells in PK 11195-treated P19 stem cells that may represent both cellular debris and dead cells (Figure 4A). The cell mass is represented in Figure 4C. After 72 and 96 h of treatment, the cell density was significantly decreased in P19 stem cells treated with RA or PK 11195 compared to undifferentiated P19 stem cells (Figure 4C, *p* < 0.001). Notably, the cell density was lower in RA-treated cells than in PK 11195-treated cells at 72 (Figure 4C, *p* < 0.001) and 96 h (Figure 4C, *p*< 0.01) of culture.

### 3.5. Cell Death during Cell Differentiation of P19 Stem Cells

**Figure 5:**
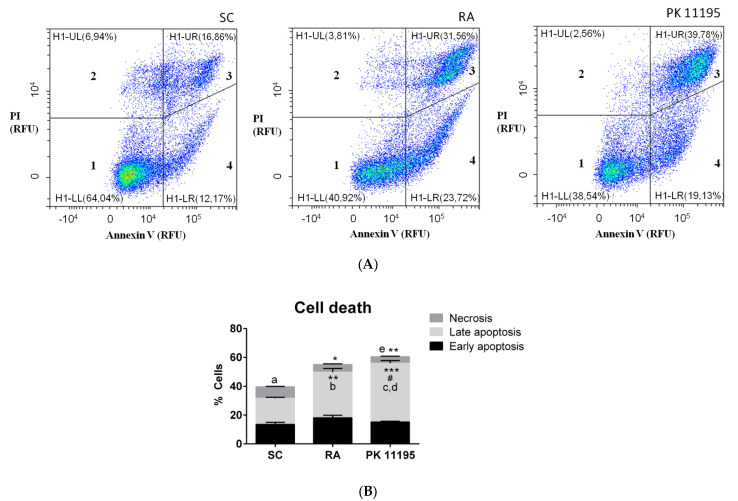
Cell death types in undifferentiated P19 stem cells (SC) and cells treated with 1 μM retinoid acid (RA) or 50 μM PK 11195 (PK 11195) during 4 days of treatment. (**A**) Cell death was analyzed by flow cytometry using propidium iodide (PI) together with Annexin V. Representative histograms show relative units of fluorescence (RFU) of PI and/or Annexin V for each one of the experimental conditions (SC, RA, and PK 11195). 1= Live cells; 2= Necrotic cells; 3= Late apoptotic cells; 4= Early apoptotic cells. (**B**) Bar chart shows means ± SEM from the percentage of necrotic, early apoptotic and late apoptotic cells from three independent cell cultures and treatments. * *p* < 0.05 *versus* SC; ** *p* < 0.01 *versus* SC. *** *p* < 0.001 *versus* SC; # *p* < 0.05 *versus* RA; ^a^ *p* < 0.05 Necrosis *versus* late apoptosis in SC; ^b^ *p* < 0.05 Late apoptosis *versus* necrosis and early apoptosis in RA; ^c^ *p* < 0.05 Late apoptosis *versus* early apoptosis in PK 11195; ^d^ *p* < 0.01 Late apoptosis *versus* necrosis in PK 11195; ^e^ *p* < 0.05 Necrosis *versus* early apoptosis in PK 11195.

Given that PK 11195 can regulate cell death under neurodegeneration and neurodifferentiation processes [2,3,4,5,6,7], the types of cell death observed during the differentiation of P19 stem cells are presented in Figure 5 (A, B). The percentage of necrotic cells was significantly lower in P19 cells treated with RA (Figure 5B, *p* < 0.05) or PK 11195 (Figure 5B, *p* < 0.01) compared to undifferentiated P19 stem cells (SC). However, the percentage of late apoptotic cells was significantly higher in RA-treated cells (Figure 5B, *p*< 0.01) and PK 11195-treated cells (Figure 5B, *p* < 0.001) compared to undifferentiated P19 stem cells. The percentage of cells in late apoptosis was also higher in PK 11195-treated cells than in RA-treated cells (Figure 5B, *p* < 0.05). Additionally, lower percentage of necrotic cells than late apoptotic cells was detected in undifferentiated P19 stem cells (Figure 5B, *p* < 0.05). The RA-treated cells showed the highest percentage of cells in late apoptosis compared to necrotic or early apoptotic cells (Figure 5B, *p*< 0.05). The PK 11195-treated cells also exhibited an increased percentage of late apoptotic cells compared to necrotic cells (Figure 5B, *p*< 0.01) and early apoptotic cells (Figure 5B, *p*< 0.05). Additionally, the percentage of necrotic cells in PK 11195-treated cells was lower than the percentage of early apoptotic cells (Figure 5B, *p* < 0.05).

### 3.6. Mitochondrial Function during the Differentiation of P19 Stem Cells

**Figure 6:**
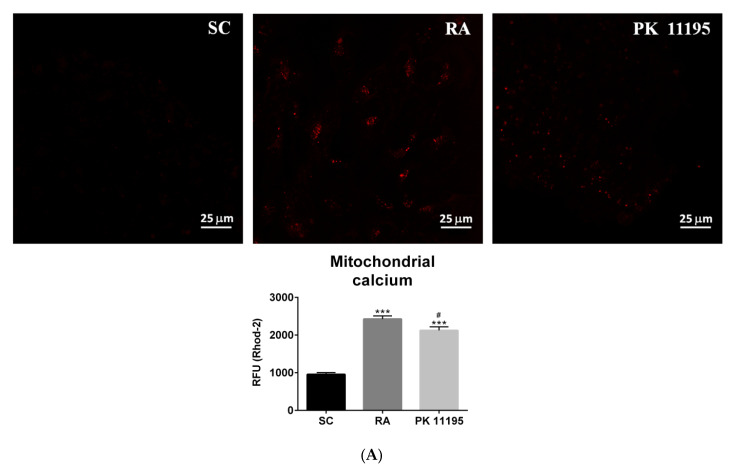

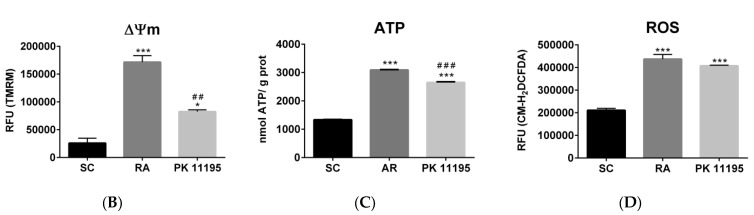
Mitochondrial parameters in undifferentiated P19 stem cells (SC) and cells treated with 1 μM retinoid acid (RA) or 50 μM PK 11195 (PK 11195) during 4 days of treatment. (**A**) Mitochondrial Ca^2+^ was assayed by fluorescence of Rhod-2 and representative micrographs of Rhod-2 fluorescence are shown for each one of the experimental conditions (SC, RA, and PK 11195). Bar chart shows quantification of the mitochondrial Ca^2+^ represented as means ± SEM from three independent cell cultures and treatments. Scale bar at the bottom right, 25 μM. (**B**) The mitochondrial membrane potential (∆Ψm) was analyzed by fluorescence of TMRM. Bar chart shows means ± SEM of the ∆Ψm from three independent cell cultures and treatments. (**C**) The ATP content was evaluated by bioluminescence. Bar chart shows the quantification of the intracellular ATP levels (nmol/g protein) represented as means ± SEM from three independent cell cultures and treatments. (**D**) Reactive oxygen species (ROS) levels were analyzed by fluorescence of CM-H_2_DCFDA. Bar chart shows the quantification of the intracellular ROS levels represented as means ± SEM from three independent cell cultures and treatments. * *p* < 0.05 *versus* SC; *** *p* < 0.001 *versus* SC; # *p* < 0.05 *versus* RA; ## *p* < 0.01 *versus* RA; ### *p* < 0.001 *versus* RA. RFU, Relative units of fluorescence.

Mitochondrial functions are key for cell differentiation and PK 11195 can affect several ones—including ROS generation, mitochondrial Ca^2+^ levels, ATP synthesis, and the ∆Ψm [2,3,4,5,6,7]—processes that may be involved in its neurodifferentiation potential. In this way, we have assayed all these parameters in our experimental conditions (SC, RA, PK 11195), as is shown in Figure 6. Mitochondrial Ca^2+^ was detected using fluorescence microscopy with the Rhod-2 dye (Figure 6A). Treatment of P19 stem cells with RA or PK 11195 increased the mitochondrial Ca^2+^ levels compared to undifferentiated P19 stem cells (SC, Figure 6A, *p* < 0.001). However, PK 11195-treated cells showed lower mitochondrial Ca^2+^ levels than RA-treated cells (Figure 6A, *p* < 0.05). Representative micrographs of Rhod-2 fluorescence also revealed mitochondrial Ca^2+^ clusters in RA-treated cells, while mitochondrial Ca^2+^ seemed to be more dispersed throughout the cytosol in PK 11195-treated cells (Figure 6A). The ∆Ψm, as assayed by measuring the fluorescence of the TMRM dye, was also significantly increased in P19 stem cells treated with RA (*p*< 0.001) or PK 11195 (*p* < 0.05) compared to undifferentiated P19 stem cells (Figure 6B). Notably, the ∆Ψm was much lower in PK 11195-treated cells than in RA-treated cells (Figure 6B, *p* < 0.01). The ATP content was evaluated by bioluminescence. The intracellular levels of ATP were significantly increased in P19 stem cells treated with RA or PK 11195 (Figure 6C, *p*< 0.001) compared to undifferentiated P19 stem cells. The ATP content was also lower in PK 11195-treated cells than in RA-treated cells (Figure 6C, *p* < 0.001). Finally, the generation of intracellular ROS was analyzed by detecting the fluorescence of CM-H_2_DCFDA (Figure 6D). Treatment of P19 stem cells with RA or PK 11195 significantly increased the ROS levels compared to undifferentiated P19 stem cells (Figure 6D, *p*< 0.001).

## 4. Discussion

The recovery of lost neurons by inducing neuronal differentiation of transplanted stem cells is one of the most promising therapeutic strategies in response to a neurogenerative process after an injury in the CNS [20,21]. TSPO plays a relevant role under neuropathological conditions, by regulating cell death, cell proliferation and cell differentiation processes, among others (e.g., ATP production, Ca^2+^ levels, ROS generation, ∆Ψm, etc.) [2,3,4,5,6,7]. In the present work, we show that the treatment of pluripotent P19 stem cells with the TSPO ligand, PK 11195, activates cell differentiation by inducing cell extensions (similar to P19 cells exposed to RA), but also robust formation of spherical growing colonies that resemble embryoid bodies. This effect of PK 11195 on inducing cell differentiation has been previously observed in PC12 and N1E-115 cells, where this TSPO ligand was able to induce neuronal differentiation [7,19]. TSPO ligands of the new generation, such as MGV-1, are also able to induce the neuronal differentiation of PC12 cells in vitro [13]. However, to date, the effect of TSPO ligands on inducing cell differentiation of embryonic pluripotent stem cells has not been described. Interestingly, differentiated P19 stem cells always showed a loss of TSPO protein expression after treatment with PK 11195 or RA. Therefore, TSPO appears to exert a repressive effect on the differentiation of P19 stem cells, since its presence is associated with an undifferentiated and pluripotent state. Consistent with our data, the TSPO protein was previously described to be expressed at low levels in neuronal and progenitor stem cells in neurogenic zones (e.g., hippocampus and subventricular zones) in the healthy brain [27], while its levels decrease during the neuronal differentiation of these stem/progenitor cells [8,28].

The process of differentiation of P19 stem cells induced by PK 11195 was similar in many aspects to that observed in RA-treated cells, since both treatments decreased cell proliferation (cell density was noticeably low at 72 and 96 h of culture), increased apoptotic cell death (1.25- (RA-treated cells) and 1.5- fold (PK 11195-treated cells) compared to SC cells) and induced a remodeling in the cell cycle. Embryonic stem cells exhibit a special cell cycle structure that is characterized by short G_1_ and G_2_ phases and by a high proportion of cells in S phase [29], as we observed in our undifferentiated P19 stem cells. However, the cell cycle shifted to the canonical cycle in differentiated P19 stem cells treated with PK 11195 or RA, since cultures showed a higher proportion of cells in G_0_/G_1_ phase and shorter S phase. Likewise, the apoptosis machinery is altered in stem cells to trigger mitotic signals [30], and thus the differentiation of stem cells seems to require cell death signals, as we also observed in our present study. Apoptosis activation has already been described during the RA-induced differentiation of P19 stem cells into neurons or cardiomyocytes [31,32]. Likewise, differentiated P19 stem cells with RA are more susceptible to mitochondrial cell death [33,34]. Notably, micromolar concentrations of TSPO ligands exert antiproliferative effects and activate mitochondrial cell death in different cell types [5,12]. Therefore, the high TSPO levels observed in undifferentiated P19 stem cells would be a key target to decrease cell proliferation and induce apoptosis, thus leading to the observed modulation of the cell cycle to favor cell differentiation processes.

According to previous studies, undifferentiated P19 stem cells have round, quiescent and weakly polarized mitochondria that are mainly located close to nuclei [33,35]. RA activates mitochondria during the differentiation of P19 stem cells, so that mitochondria present a longer and filamentous morphology, greater cristae development, interconnectivity, and dispersion throughout the cytoplasm [33,35]. Accordingly, our treatments with RA induced a major mitochondrial connectivity during the differentiation of P19 stem cells, while treatment with PK 11195 induced a greater dispersion of mitochondria throughout the cytoplasm. Thus, mitochondrial function is a key for differentiation of pluripotent P19 stem cells, since undifferentiated P19 stem cells have a preference for glycolytic metabolism, compared to RA-treated P19 stem cells, which have high glycolytic rates but also utilize oxidative phosphorylation [33]. In this way, we also observed mitochondrial activation in P19 stem cells treated with RA or PK 11195, since the ∆Ψm was increased, as reflected by higher mitochondrial polarization. Consequently, ROS generation, mitochondrial Ca^2+^ levels and ATP content were significantly increased in differentiated P19 stem cells. ROS and Ca^2+^ signaling are crucial for the physiological cell differentiation of pluripotent stem cells and adult stem cells, including neural stem cells [35]. Increased intramitochondrial Ca^2+^ levels positively affect energy metabolism through the stimulation of ATP production by oxidative phosphorylation (OXPHOS) [35].

However, the excessive accumulation of Ca^2+^ in the mitochondria also leads to apoptosis by inducing the opening of the permeability transition pore and the subsequent release of cytochrome c into the cytoplasm [35]. Notably, micromolar concentrations of PK 11195 induce the opening of the mitochondrial permeability transition pore and produce collapse of the ∆Ψm, thus increasing the susceptibility to mitochondrial cell death [5,12]. This finding may explain why PK 11195 caused a lower increase in the ∆Ψm in P19 stem cells than RA. In addition, TSPO is also involved in the generation of ROS [5,12]. Oxidative stress promotes the peroxidation of cardiolipin during the RA-induced differentiation of P19 stem cells [36]. Cardiolipin peroxidation is also a key step in the mitochondrial cell death pathway driven by TSPO [5,12]. Therefore, ROS generation may be associated with a greater susceptibility of PK 11195-treated cells to cell death and is also a relevant factor for inducing cell differentiation processes. Consistent with our data, previous studies reported alterations in the expression of differentiation markers in RA-treated P19 cells, when they were differentiated in the presence of N-acetylcysteine, since this antioxidant maintains P19 cells in a trophoectodermal stage [33].

PK 11195 and RA share common features in their capacity to induce the differentiation of P19 stem cells, but with specific differences in their mechanism of action. RA activates the differentiation of pluripotent stem cells by directly targeting specific nuclear retinoid acid receptors (RARs) that are known to be nuclear transcription factors. Once activated by RA, RARs regulate the expression of RA-target genes (e.g., homeobox genes) by consensus sequences located on their promoters known as retinoic acid response elements (RARE), leading to changes in cell differentiation, cell proliferation, and apoptosis. Three RARs subtypes have been identified (RARα, RARβ, and RARγ) in P19 stem cells [36,37]. However, TSPO appears to be part of a retrograde signaling pathway from mitochondria to the nucleus that is associated with the canonical pathway for modulating nuclear gene expression in order to affect cell viability, cell proliferation, cell death, migration, etc., including the development of neurons, microtubule dynamics, and the formation of cellular protrusions [7]. This signaling pathway is driven by mechanisms that include ∆Ψm collapse, ROS generation, increased Ca^2+^ levels, and ATP production, and all of these mechanisms are activated by micromolar concentrations of PK 11195 [7], as we also observed in our present study. Thus, this mitochondria-to-nucleus signaling related to TSPO may support the effect of PK 11195 on inducing differentiation of P19 stem cells. Accordingly, PK 11195 produced transcriptional changes in protein markers of pluripotency and neuronal differentiation in P19 stem cells, which were also observed in RA-treated cells.

P19 stem cells grown in a monolayer and treated with 1μM RA yielded a mixed population of differentiated cells with both endodermal (Troma-1 expression) and neuroectodermal (expression of Nestin, BLBP, Msi1, Mash1, Tbr2, NeuroD1, Dcx, NeuN, and Tubb3 markers) characteristics after 4 days of culture, as previously described [22,33,36,37]. Interestingly, PK 11195-treated cells also exhibited transcriptional changes in protein levels after 4 days of culture, since we observed a significant loss of the expression of pluripotency markers (Oct4-A, Nanog, and Sox-2), although at a lower level than in RA-treated cells. P19 stem cells treated with PK 11195 also showed expression of markers for neuronal/multipotent stem cells (Nestin and Msi1) and radial glia cells (BLBP), with a minimal expression of endodermal markers (Troma-1). Likewise, PK 11195-treated cells expressed markers of neuronal progenitors (Mash1 and Tbr2) at higher levels than RA-treated cells. Neurogenesis was observed by detecting the expression of Dcx and NeuroD1, since both were expressed in P19 cells treated with RA or PK 11195 [24]. NeuroD1 is a marker for the identification of mitotic neuronal cells, while Dcx has been used to label postmitotic neuronal precursor cells and immature neurons [24]. Only NeuroD1 expression showed differences between treatments, since NeuroD1 was observed at higher levels in RA-treated cells.

We should note that different developmental grades of early postmitotic neurons have been detected by measuring the expression of NeuN and Tubb3 [24,38]. Notably, PK 11195-treated cells showed much higher levels of Tubb3 expression, while RA-treated cells showed increased expression of NeuN. Tubb3 is a neuron-specific class III β-tubulin that mainly localizes in neural axons [38], thus revealing important processes of neurite development in PK 11195-treated cells, as were observed microscopically. Although NeuN is commonly localized in neuronal soma, recent studies have reported its cytoplasmic localization in several peripheral tissues—such as heart, liver, lung, and kidneys—where NeuN has functions other than neuronal differentiation, specifically those related to RNA splicing [39]. Expression of NeuN in undifferentiated P19 stem cells supports these non-neural functions of NeuN. Importantly, undifferentiated P19 stem cells also expressed some markers of neuronal stem/progenitor cells (mainly Tbr2). Accordingly, previous studies have already described the spontaneous expression of neuronal markers in undifferentiated human mesenchymal stem cells from different sources (e.g., adipose tissue, skin, periodontal ligament, and dental pulp), with unclear biological significance [40].

## 5. Conclusions

Our present data reveal the expression of TSPO protein in association with a pluripotent state in P19 stem cells. Likewise, we observed an effect of the TSPO ligand, PK 11195, on inducing the differentiation of these pluripotent P19 stem cells with a strong predisposition toward neuronal lineages compared to the cell differentiation induced by RA. P19 stem cells treated with PK 11195 showed robust development of embryoid bodies with a clearly predetermined neural destination, as evidenced by high levels of neuronal progenitors (Mash1 and Tbr2), immature neurons (NeuroD1) and overall, the high level of expression of a marker for axonal development (Tubb3). However, classic treatment with RA induced higher expression of markers of neuronal/multipotent stem cells (Nestin and Msi1) and immature neurons (NeurD1), as well as higher expression of markers that may reflect endodermal destinies (Troma-1 and NeuN). As we observed in RA-treated cells, PK 11195-induced neuronal differentiation is accompanied by the changes in the cell cycle, decreases in cell proliferation and activation of cell death. Mitochondrial metabolism was also activated so that we observed increases in the levels of ROS, Ca^2+^, and ATP, as well as changes in the ∆Ψm in PK 11195-treated P19 stem cells. Mitochondrial activation induced by PK 11195 by targeting the TSPO can affect proteins expression related to pluripotency and neurogenesis and activate neuronal differentiation of pluripotent P19 stem cells (see graphical abstract).

## Data Availability

All relevant data are presented in this paper.

## Supplementary Materials

The following are available online at https://www.mdpi.com/article/10.3390/cells10102784/s1; Supplementary Figure S1: Verification of specificity of the different primary antibodies used in the present study.
