# Supplementary material for: Neurogenic Potential of the 18-kDa Mitochondrial Translocator Protein (TSPO) in Pluripotent P19 Stem Cells
Source: Cells. 2021 Oct 17;10(10):2784. doi: 10.3390/cells10102784 (PMC8534396; doi:10.3390/cells10102784)
Supplement: Supplementary file 1 [file cells-10-02784-s001.zip › cells-1330544-supplementary.pdf]

TSPO

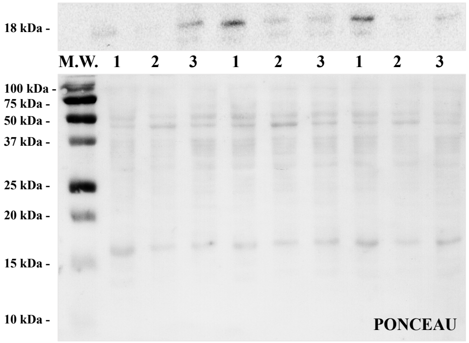

1, Undiferentiated P19 stem cells (SC)
2, RA-treated P19 stem cells (RA)
3, PK 11195-treated P19 stem cells (PK 11195)

OCT4-A

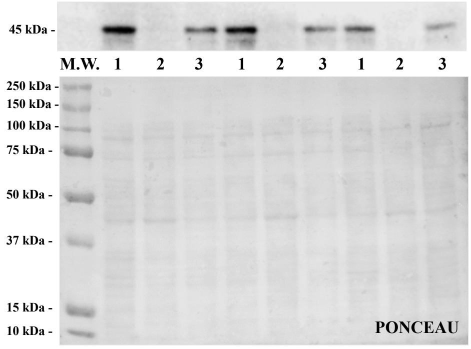

1, Undiferentiated P19 stem cells (SC)
2, RA-treated P19 stem cells (RA)
3, PK 11195-treated P19 stem cells (PK 11195)

NANOG

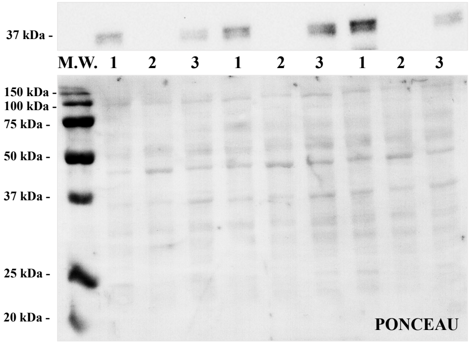

1, Undiferentiated P19 stem cells (SC)
2, RA-treated P19 stem cells (RA)
3, PK 11195-treated P19 stem cells (PK 11195)

SOX-2

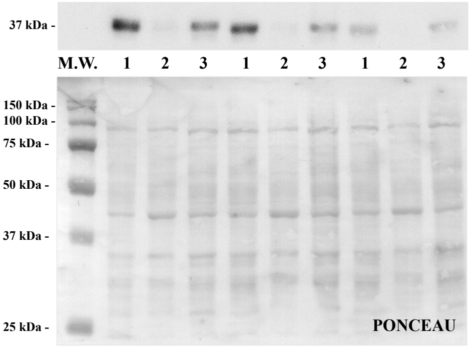

1, Undiferentiated P19 stem cells (SC)
2, RA-treated P19 stem cells (RA)
3, PK 11195-treated P19 stem cells (PK 11195)

TROMA-1

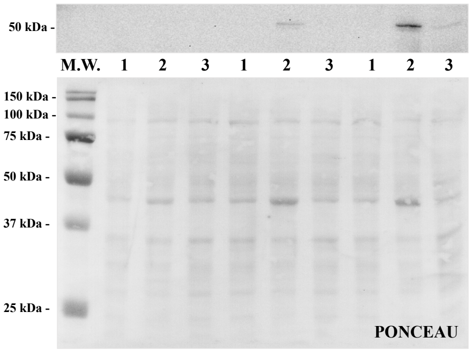

1, Undiferentiated P19 stem cells (SC)
2, RA-treated P19 stem cells (RA)
3, PK 11195-treated P19 stem cells (PK 11195)

NESTIN

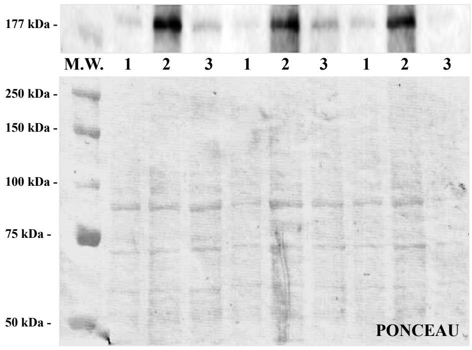

1, Undiferentiated P19 stem cells (SC)
2, RA-treated P19 stem cells (RA)
3, PK 11195-treated P19 stem cells (PK 11195)

BLBP

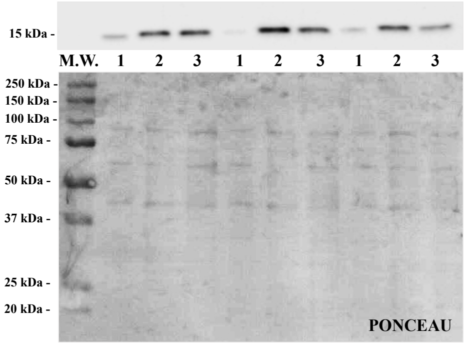

1, Undiferentiated P19 stem cells (SC)
2, RA-treated P19 stem cells (RA)
3, PK 11195-treated P19 stem cells (PK 11195)

MSI1

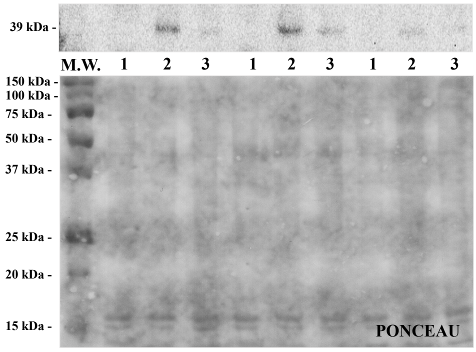

1, Undiferentiated P19 stem cells (SC)
2, RA-treated P19 stem cells (RA)
3, PK 11195-treated P19 stem cells (PK 11195)

MASH1

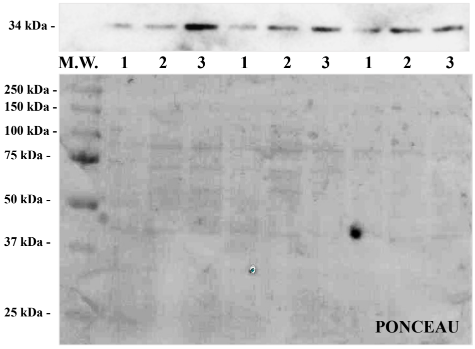

1, Undiferentiated P19 stem cells (SC)
2, RA-treated P19 stem cells (RA)
3, PK 11195-treated P19 stem cells (PK 11195)

TBR2

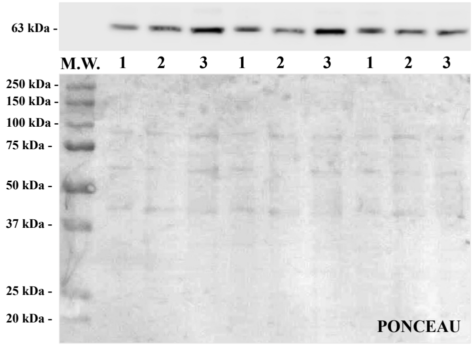

1, Undiferentiated P19 stem cells (SC)
2, RA-treated P19 stem cells (RA)
3, PK 11195-treated P19 stem cells (PK 11195)

NEUROD1

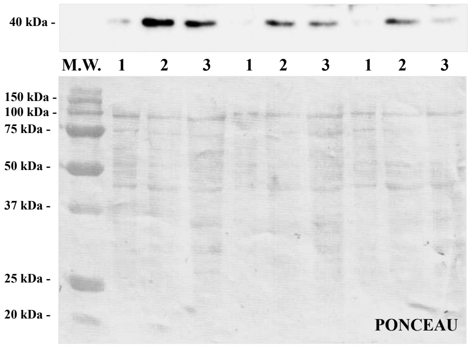

1, Undiferentiated P19 stem cells (SC)
2, RA-treated P19 stem cells (RA)
3, PK 11195-treated P19 stem cells (PK 11195)

DCX

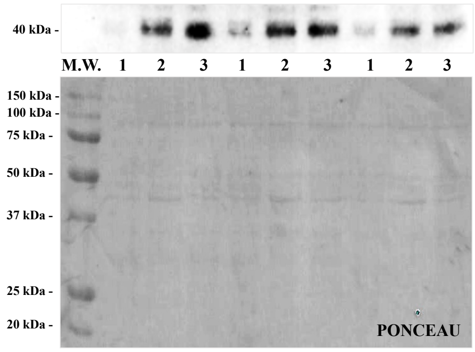

1, Undiferentiated P19 stem cells (SC)
2, RA-treated P19 stem cells (RA)
3, PK 11195-treated P19 stem cells (PK 11195)

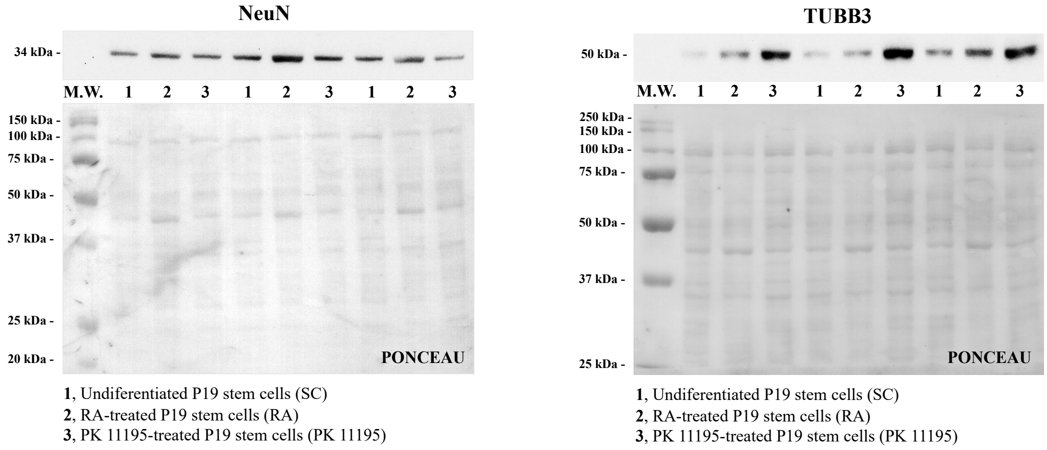

Supplementary figure S1. Verification of specificity of the different primary antibodies used in the present study. Figure S1 shows representative western blots from undifferentiated P19 stem cells (1) as well as P19 cells treated with retinoid acid (2) or PK 11195 (3) marked with the different primary antibodies of the study (TSPO, Oct- 4A, Nanog, Sox-2, Troma-1, Nestin, BLBP, Msi1, Mash1, Tbr2, NeuroD1, Dcx, NeuN and Tubb3). The Ponceau red staining of each PVDF membrane is also shown for each primary antibody in order to demonstrate equal protein loading. M.W., Molecular Weight.
